# Response to Letter to the Editor

**DOI:** 10.36469/001c.74215

**Published:** 2023-04-12

**Authors:** Folashayo Adeniji, Taiwo Obembe

**Affiliations:** 1 Department of Health Policy & Management, College of Medicine University of Ibadan, Ibadan, Nigeria

**Keywords:** catastrophic health expenditure, sub-Saharan Africa, cardiovascular diseases, health economics

## Abstract

The authors respond to the comments raised in the letter regarding Adeniji and Obembe’s article on catastrophic health expenditures in sub-Saharan Africa.

We thank Ishir Narayan for his interest and valuable comments on our recently published article. Regarding the variables that were controlled for in the model that predicted the risk of higher catastrophic health expenditures (CHE), it is well known that “diabetes, hypertension, truncal obesity, dyslipidemia,” and variables such as tobacco use, excessive alcohol consumption, low intake of fruits, lack of physical activities are risk factors for CHE and many other chronic noncommunicable diseases.

Among the disease entities reported in the data used for the study, we noted that hypertension and angina were used to depict the presence of CVD. Hence, it was methodologically impossible to introduce, for instance, hypertension as one of the predictor variables/covariates. Instead, we captured the presence of other health conditions that were reported in the data to see their impact on the experience of CHE. This plausible approach was noted in our methods. Information on the presence of diabetes, truncal obesity, and dyslipidemia were not reported in the data, rendering it impossible to have controlled for those conditions as risk factors for CHE or CVD.

As to whether households that reported having CVDs had higher risk of CHE across all thresholds, we believe this assertion is correct because more households with CVDs reported higher CHE across all thresholds (see **Table 5**). While this was significant in some cases, it was not in others. In fact, despite the width of the confidence interval, at the 25% threshold, the differences between households with and households without CVD in Ghana was significant at *P* < .01. Also, having insurance cannot be a risk factor for CHE. Theoretically, having insurance is meant to prevent the occurrence of CHE among individuals and households since it is a means for financial protection against excessive healthcare spending.

Narayan’s comment on the need for a consensus in terms of the ideal thresholds to be utilized when estimating CHE is valid to the best of our understanding. That is why it is always advisable to adopt a sensitivity analysis by implementing different thresholds, as we did in the paper. Alternatively, the rank-dependent threshold has been recommended by Ataguba.^1^ This was adopted in one of our recent articles.^2^

Overall, our findings contribute to the ongoing conversation on universal health coverage in sub-Saharan Africa, especially for individuals and households with chronic conditions. However, we noted that more research needs to be conducted in this regard and highlighted the need for data to conduct such studies in the subregion.

Folashayo Ikenna Peter Adeniji

Taiwo Akinyode Obembe

Department of Health Policy & Management, College of Medicine

University of Ibadan, Ibadan, Nigeria
